# A Transcriptional Analysis of Cattle Immune Cells Reveals a Central Role of Type 1 Interferon in the In Vitro Innate Immune Response against *Mycobacterium bovis*

**DOI:** 10.3390/pathogens12091159

**Published:** 2023-09-14

**Authors:** Federico Carlos Blanco, María Mercedes Bigi, Elizabeth Andrea García, María Teresa Elola, Cristina Lourdes Vázquez, Fabiana Bigi

**Affiliations:** 1 Instituto de Agrobiotecnología y Biología Molecular (IABIMO), INTA-CONICET, N. Repetto and De los Reseros, Buenos Aires 1686, Argentina; blanco.federico@inta.gob.ar (F.C.B.); garcia.elizabeth@inta.gob.ar (E.A.G.);; 2 Instituto de Biotecnología, CICVyA, Instituto Nacional de Tecnología Agropecuaria, N. Repetto and De los Reseros, Buenos Aires 1686, Argentina; 3 Instituto de Investigaciones Biomédicas (UBA-CONICET), Facultad de Medicina, Universidad de Buenos Aires, Buenos Aires 1417, Argentina; mbigi@fmed.uba.ar; 4 Instituto de Química y Fisicoquímica Biológicas Prof. Dr. Alejandro Paladini (UBA-CONICET), Facultad de Farmacia y Bioquímica, Universidad de Buenos Aires, Buenos Aires 1113, Argentina

**Keywords:** bovine tuberculosis, *Mycobacterium bovis*, type I interferon, KEAP1-NFE2L2, bovine macrophages

## Abstract

Bovine tuberculosis is a chronic infectious disease primarily caused by *Mycobacterium bovis*, a bacterium that affects cattle and other mammals, including humans. Despite the availability of vast research about the immune response mechanisms of human tuberculosis caused by *Mycobacterium tuberculosis*, the knowledge of bovine tuberculosis’s immunology, particularly regarding the innate immune response, still remains scarce. In this study, we compared the transcriptome of cell cultures containing lymphocytes and *M. bovis* infected-macrophages with two strains of variable virulence, the virulent Mb04-303 strain and the attenuated Mb534. To that end, we infected bovine macrophages at a multiplicity of infection of one, and co-cultured the infections with autologous lymphocytes. RNA obtained from the co-cultures was sequenced to identify differentially expressed gene pathways by using the database Reactome. The RNA-seq analysis showed that the Mb04-303 infection upregulated the type 1 interferon signalling pathway, while it downregulated the KEAP1-NFE2L2 pathway. According to the literature, this last pathway is involved in the activation of antioxidant genes and inflammasome. In addition, the macrophages infected with Mb04-303 recruited more Galectin 8 than those infected with Mb534. This result indicates that Mb04-303 induced higher phagosome membrane damage, with the possible concomitant release of bacterial compounds into the cytoplasm that activates the type I signalling pathway. Altogether, Mb04-303 repressed the antioxidant and anti-inflammatory responses, likely impairing interleukin-1β activation, and trigged the canonical type 1 interferon signalling. Although these responses led to the control of bacterial replication during early infection, the virulent strain eventually managed to establish a successful infection.

## 1. Introduction

*Mycobacterium bovis* is the main causative agent of bovine tuberculosis (bTB), a chronic respiratory disease in several mammals, including wildlife reservoirs and humans. This disease mainly affects rural families in close contact with animals, although contaminated food (primarily unpasteurised dairy products or their derivatives) also represents a risk of transmission [1]. While *Mycobacterium tuberculosis*, the main agent of human tuberculosis, is capable of inducing phagosomal arrest at early stages, which leads to a non-replicative or low-replicative state of the disease, *M. bovis* leads to more severe stages, and causes an acute infection with active mycobacterial replication [2]. Thus, bTB is a direct risk to agricultural economies and a threat to human health.

The adaptive immune response characterised by interferon gamma-producing T helper cells (CD4+) is the hallmark of protection against tuberculosis. However, studies in cattle and other animal models indicate that less-studied arms of the immune response, such as the innate and trained innate immune responses, are also critical elements of this protective anti-TB and bTB response (reviewed in [3]). In these non-specific immune responses, γδ T cells, monocytes, NK cells, and alveolar macrophages play an essential role in bovine tuberculosis. In addition, γδ T cells, monocytes, and alveolar macrophages can not only rapidly respond to bovine bTB infection, but also build a non-specific immunological memory (reviewed in [3]).

The induction of trained immunity in mycobacterial infections hinges on a specific mechanism. This process initiates with the interaction between the pathogen and the intracellular receptor NOD2. Intriguingly, this interaction requires the internalization of factors released by the bacteria [3].

Additionally, the innate immune response involves critical players such as γδ T cells and NK cells. In the early stages of infection, γδ T cells actively secrete vital cytokines like IFN-γ, IL-10, and IL-17A, along with chemokines. This secretion endows these cell populations a pivotal role, influencing the subsequent adaptive immune response towards a Th1 bias [3].

Furthermore, natural killer (NK) cells serve as a bridge connecting the innate and adaptive immune responses. Within draining lymph nodes, NK cells significantly contribute to the early innate response against bacterial infections by producing interferon-gamma (IFN-γ) [4]. These effector responses rely heavily on interactions with antigen-presenting cells (APCs), particularly dendritic cells (DCs).

A noteworthy aspect of NK cells is their direct cytotoxic effect against macrophages infected with mycobacteria, which underscores their multifaceted role in the immune defence against these pathogens [4].

As in human tuberculosis, the outcome of *M. bovis* infections depends on the immune background of the individual host and the genomics and phenomics variability of the *M. bovis* strains. In a previous study, we found that the magnitude of the innate immune response induction depends on the secretome of *M. bovis* strains with different virulence levels [4]. In the case of the highly virulent strain Mb04-303, either the bacterium itself or its secreted compounds induce a powerful innate immune response capable of controlling *M. bovis* replication inside bovine macrophages [4].

To decipher the mechanisms underlying an innate response that effectively controls the replication of *M. bovis* within bovine macrophages, we assessed the transcriptional signatures that the virulent strain Mb04-303 induces in immune cells. Here, Mb04-303 infections induced upregulation of IFN type I gene pathways. Conversely, the attenuated strain failed to induce an effective innate response and induced higher expression of the KEAP1-NFE2L2 pathway, a signature of antioxidant and anti-inflammatory responses [5]. The impact of antioxidant anti-inflammatory mechanisms triggered by NFE2L2 on *M. tuberculosis* infection is still unclear. However, although NFE2L2 may act by protecting alveolar macrophages during *M. tuberculosis* infection, it unfavourably affects the early control of intracellular bacterial replication [6].

## 2. Materials and Methods

### 2.1. Bacterial Strains and Culture Media

The *M. bovis* strains were grown in a Middlebrook 7H9 medium (Becton Dickinson, Holdrege, NE, USA), supplemented with 0.5% bovine serum albumin fraction V (BSA) (Sigma-Aldrich, St. Louis, MO, USA), 0.4% glucose and 0.4% pyruvate (Anedra, Research AG, Buenos Aires, Argentina) (ADP) with or without 0.05% Tween 80 (Sigma-Aldrich, St. Louis, MO, USA) or Middlebrook 7H10 Agar Base (Becton Dickinson, Holdrege, NE, USA) supplemented with ADP. When necessary, 20 μg/mL of kanamycin (Sigma-Aldrich, St. Louis, MO, USA) was added into the media. *M. bovis* strains were engineered to express RFP (red fluorescent protein) constitutively, and were grown under the same conditions as the other strains.

### 2.2. Sample Collection

Blood samples were collected from healthy adult calves (2–3 years of age) via jugular vein puncture. The animals were crossbred calves (Hereford and Aberdeen Angus) from a herd settled in an INTA field with no history of bTB and paratuberculosis within the last 5 years. The animals were negative to bTB or paratuberculosis according to an IFN-γ ELISA assay and tuberculin skin test. The sampling was performed in compliance with the regulations of the Ethical Committee of INTA (CICUAE, preapproved protocol: Bo1).

### 2.3. M. bovis Preparation for Cell Infection

*M. bovis* strains were cultured until obtaining an exponential growth phase, and then harvested. The harvested bacteria were washed to eliminate traces of culture medium and resuspended in an RPMI (Invitrogen, Thermo Fisher Scientific, Waltham, MA, USA) medium. The bacterial suspensions were passed through a syringe needle (25 gauge) to disaggregate bacteria clumps. The remaining clumps were removed via a low-speed centrifugation for 10 min. The bacterial suspension concentration was determined by measuring the optical density OD_600nm_ of the supernatant. The multiplicity of infection (MOI) of all *M. bovis* strains was adjusted to 1 to perform the infections.

### 2.4. Mononuclear Cell Isolation, Co-Cultures and Infections

Heparinised whole blood samples (50 mL) from different animals were used to isolate peripheral blood mononuclear cells (PBMCs) using Histopaque 1077 (Sigma-Aldrich, St. Louis, MO, USA), according to the manufacturer’s protocol. PBMCs were seeded under different conditions depending on the type of the experiment. For the co-cultured assays, cells were seeded for 24 h in 12-well tissue culture plates using an RPMI complete medium supplemented with 10% autologous plasma and 1× Antibiotic-Antimycotic (Anti Anti) (Invitrogen, Thermo Fisher Scientific, Waltham, MA, USA). Then, the non-adherent cells were removed and cultured separately, whereas the adherent cells were incubated with 10% autologous plasma for 5 days at 37 °C in an atmosphere of 5% CO_2_ to obtain differentiated macrophage.

Macrophages were infected with Mb534 or Mb04-303 at a MOI of 1. After 3 h of infection (bacterial uptake), the macrophages were washed with PBS and co-cultivated with the autologous non-adherent cells for 16 h. The non-adherent cells from the infected co-cultures were pelleted and resuspended in 1 mL of Trizol (Invitrogen, Thermo Fisher Scientific, Waltham, MA, USA). The macrophages were detached using the same aliquot of Trizol, and finally, the samples were stored at −80 °C until RNA purification.

For the mycobacterial intracellular replication assay, PBMCs were seeded in 24-well tissue culture plates using an RPMI complete medium supplemented with 10% autologous plasma and 1× Anti Anti. After 5 days of differentiation, the cells were infected with the different strains in triplicate for 3 h (bacterial uptake) and at an MOI of 1. Then, the cells were washed three times with PBS, and the infected macrophages were incubated for 72 h with or without autologous lymphocytes at 37 °C and 5% CO_2_. The cells were lysed with triton X-100, and seeded in 7H10 Agar Base (Becton Dickinson, Holdrege, NE, USA) supplemented with ADP. Colony forming units (CFUs) were counted after 21 days for all the evaluated experimental conditions.

For the immunofluorescence assays, PBMCs were seeded into 24-well plates containing untreated glass coverslips using an RPMI complete medium supplemented with 10% autologous plasma and 1× Anti Anti. After 5 days of differentiation, the macrophages were infected with Mb04-303-RFP (red fluorescent) or Mb534-RFP (red fluorescent) at an MOI of 1 and incubated for 3 h at 37 °C and 5% CO_2_ (bacterial uptake). Then, the cells were washed three times with PBS, and incubated for 24 h with RPMI complete medium supplemented with 10% autologous plasma plus Gentamicin 10 μg/mL (Sigma-Aldrich, St. Louis, MO, USA). The infected cells were fixed with 4% paraformaldehyde solution in PBS (PFA) for 30 min, and saved in PBS at 4 °C until the immunofluorescence assays were performed. Three independent infections were performed for each assay.

### 2.5. RNA Purification, Quantification and Sequencing

Total RNA from the infected co-cultures was obtained following the manufacturer’s protocol with minor modifications. Briefly, cells lysed in 1 mL of Trizol were mixed with 0.2 mL of chloroform, and then centrifuged at 12,000× *g* at 4 °C for 15 min. The aqueous phase was transferred to a new tube and mixed again with 0.1 mL of chloroform, and centrifuged at 12,000× *g* at 4 °C for 5 min. Finally, the RNA was precipitated from the aqueous phase by adding 0.6 mL of isopropanol and 60 μL of 3 M sodium acetate (Sigma-Aldrich, St. Louis, MO, USA). The RNA solution was incubated overnight at −70 °C, and then centrifuged at 12,000× *g* at 4 °C for 30 min. The pellet was washed with ethanol 70% and the purified RNA was resuspended in 20 μL DEPC water (Sigma-Aldrich, St. Louis, MO, USA). RNA quality and quantity were determined in the Bioanalyzer (Agilent, Santa Clara, CA, USA) instrument using Agilent RNA 6000 Nano Kit. The purified method yielded high quality electropherograms, and the mean value for the RNA integrity Number (RIN) of the six samples was 8.25.

Libraries for RNA sequencing were constructed using the TruSeq Stranded mRNA kit (Illumina, San Diego, CA, USA), and sequenced in the NextSeq500 (Illumina, San Diego, CA, USA) equipment from the Hospital de Niños R. Gutierrez with a 75-cycles Single End kit and 20 M readings per replicate.

### 2.6. RNA-Sequencing Analysis

De novo transcriptome reconstruction RNA-Seq was carried out on a Galaxy open-source data analysis platform (https://galaxyproject.org/ (accessed on 26 July 2022)) [7]. The Galaxy’s workflow was used to process RNA-Seq data, following the step of the tutorial previously described by Bérénice Batut et al. [8]. Briefly, the FastQC tool (Galaxy version 0.25.1 + Galaxy0) was used as quality control of FASTQ files, whereas Cutadapt (Galaxy version 4.0 + galaxy0) was used to trim and filter adapter sequences, with the following parameters: single-end reads, a minimum length (R1) of 20, and quality cutoff of 20. In addition, HISAT2 (Galaxy Version 2.2.1 + galaxy1) [9] was used to align mRNA sequencing reads to bosTau9 genome (NCBI RefSeq: GCF_002263795.1). Subsequently, StringTie (Galaxy version 2.1.72.1.7 + galaxy1) [10] was used to predict transcript structures based on the reads aligned using HISAT, whereas StringTie-merge (Galaxy version 2.1.72.1.7 + galaxy1) was used to combine redundant transcript structures across the samples and the RefSeq reference (NCBI RefSeq: GCF_002263795.1).

The transcripts of the created transcriptome were annotated using GFFcompare (version 0.11.2) [11] according to the relationship of each transcript to the RefSeq reference, and the reads per transcripts were counted using Feature Counts (Galaxy Version 2.0.1 + galaxy2) [12]. Finally, DESeq2 (Galaxy version 2.11.40.7 + galaxy1) [13] was used to generate normalised transcript counts (abundance estimates) and significance testing for differential expression, whereas Annotate DESeq2/DEXSeq (Galaxy Version 1.1.0) was used to create the output table. Data sets were deposited in GEO Submission (GSE241059).

The samples were sorted according to their adjusted *p* value, that is, the smallest false discovery rate (FDR) at which a transcript is considered significant. FDR is the expected fraction of false-positive tests among significant tests, and was calculated using the Benjamini–Hochberg multiple testing adjustment procedure.

### 2.7. Indirect Immunofluorescence

Fixed cells were incubated with a solution of PBS-NH_4_Cl 50 mM for 30 min at room temperature (RT) to quench the free aldehyde groups. Then, the cells were permeabilised with 0.05% saponin in PBS containing 1% BSA for 15 min, and incubated with the primary antibody anti-Galectin 8 diluted 1:50 in PBS, and incubated overnight in a humid chamber at RT. A secondary antibody anti-rabbit conjugated to Alexa 488 (Cell Signaling Tech., Danvers, MA, USA) was used diluted to 1:500 in PBS, and incubated for 1 h at RT. The cell nuclei were stained with TOPRO-3 (Thermo Fisher Scientific, Waltham, MA, USA; diluted 1:1000) for 30 min. Finally, the coverslips were mounted onto glass slides with Dako fluorescence mounting media (Dako, Glostrup, Hovedstaden, Denmark) and analysed via confocal microscopy. The experiments were performed in triplicates in three independent experiments.

### 2.8. Image Acquisition via Confocal Microscopy

A Leica SP5 AOBS Laser Scanning Confocal Microscope (Leica Microsystems, Wetzlar, HE, Germany) was used for the analyses. The observations were performed using a 40x/1.4 HCX-PLAPO oil objective and excitation lasers of 488, 561 and 633 nm. The parameters used were as follows: scanner frequency 200–400 Hz and PMT detectors at a scanning resolution of 1024 × 1024 pixels or 512 × 512 pixels. The same settings of the laser powers, gain, and offset were maintained for the different experiments.

### 2.9. Image Analysis

Analyses of all the images were performed using Fiji 1.49k (U.S. National Institute of Health, Bethesda, MD, USA). Fiji is based on ImageJ (available at http://fiji.sc (accessed on 10 August 2023)). The association of Galectin 8 with bacterial particles in the cells was measured by splitting the RGB image into individual channels, and subjecting the red channel (bacteria) to a pixel threshold of 50 to create a mask. The mask was subjected to “dilate tool” (1 pixel) to have a defined representation of the true outlines of mycobacteria. Then, the “Analysed particles” function of Fiji was used to measure the fluorescence intensity of Galectin 8 (which was related to the secondary antibody used to detect the primary antibody) associated with the bacteria by redirecting the measurements to the channel (green) of interest in “Set Measurements” in the Analyses function of Fiji. Fluorescence intensity values were plotted and analysed using Microsoft Excel 2016 (Microsoft) and GraphPad Prism 5 (GraphPad Software Inc., Boston, MA, USA).

### 2.10. Knockout of esxA-esxB in Mb04-303 Strain

The *esxA* and *esxB* genes were eliminated from the Mb04-303 strain by applying a method that uses the specialised phage-transduction methodology for delivering DNA to generate gene knockouts in mycobacterial species [14]. The substrate for homologous recombination consisted of a kanamycin-resistance cassette flanked by DNA segments corresponding to 200 bp of the regions upstream and downstream of the *esxB-esxA* genes. This DNA fragment, which was obtained via the polymerase chain reaction (PCR) using the primer pairs I/II and III/IV (Table S1), was inserted into the PvuI site of the pMW231 vector to interrupt the ampicillin-resistant gene. *Escherichia coli* transformant clones were selected using Luria-Bertani (LB) agar (Sigma-Aldrich, St. Louis, MO, USA) supplemented with 50 μg/mL kanamycin. The recombinant plasmid was digested with PmeI (New England Biolabs, Ipswich, MA, USA). The DNA fragment was purified several times to avoid contamination with circular plasmid, and then electroporated into the *E. coli* DY380 strain carrying the phAE87 and the ampicillin-resistant gene for homologous recombination. The λ recombination genes were previously induced by incubating 5 mL of a mid-log bacterial culture in a bath water at 42 °C for 15 min.

The transformed *E. coli* DY380 were grown on LB agar with 50 μg/mL kanamycin. The recombinant phasmid was purified and electroporated into *M. smegmatis* mc^2^ 155 to generate a transducing recombinant mycobacteriophage. Subsequently, the transduced mycobacteria were mixed with warm soft agar, plated on Middlebrook 7H10 Agar Base supplemented with 0.5% bovine serum albumin fraction V (Sigma-Aldrich, St. Louis, MO, USA), 0.4% glucose and 0.5% glycerol (GE, Boston, MA, USA) (ADG), and cultured at 30 °C. Finally, a high titre solution of the transducing mycobacteriophages was prepared and stored at 4 °C until use.

The specialised transduction was performed by mixing 1 mL of the high titres transducing phages with 50 mL of the Mb04-303 culture grown to an OD_600nm_ of 0.6–1 without Tween 80. The transduced culture was then centrifuged, washed, and resuspended in 1 mL Middlebrook 7H9 medium supplemented with ADP throughout the cell recovery time. Subsequently, the culture was plated on the Middlebrook 7H10-ADP agar containing 20 μg/mL kanamycin at 37 °C for 20–30 days.

The deletion of *esxA*-*esxB* from Mb04-303 was then confirmed via Western blot (Figure S1) and PCR using primers listed in Table S1. The Western blot analysis was performed as previously described [15]. Briefly, bacteria were incubated for 4 weeks in a BSA-free medium, and the supernatants containing secreted proteins were precipitated with 10% trichloroacetic. The proteins were resolved in SDS-PAGE and transferred to nitrocellulose membranes. The membranes were incubated with monoclonal antibodies anti-ESAT-6 and then with a secondary anti-mouse alkaline phosphatase-conjugated antibody. The Western blots were revealed after performing an incubation with 5-bromo-4-chloro-3-indolyl phosphate/nitro blue tetrazolium solution. The designated name for the mutant strain was Mb04-303ΔesxA-esxB.

## 3. Results

### 3.1. The Interferon Type 1 Signalling Pathway Is Upregulated in PBMC Infected with the Virulent Mb04-303 Strain

In a previous study, we had demonstrated that, unlike the attenuated Mb534 isolate, the virulent Mb04-303 strain induced the proliferation of interferon-gamma producer-natural killer (NK) cells when naive bovine PBMC was infected in vitro [4]. In addition, this innate immune response effectively controlled the intracellular replication of Mb04-303 in macrophages. In this paper, we used a comparative transcriptomic approach to dissect the mechanisms underlying this effective innate immune response.

**Figure 1:**
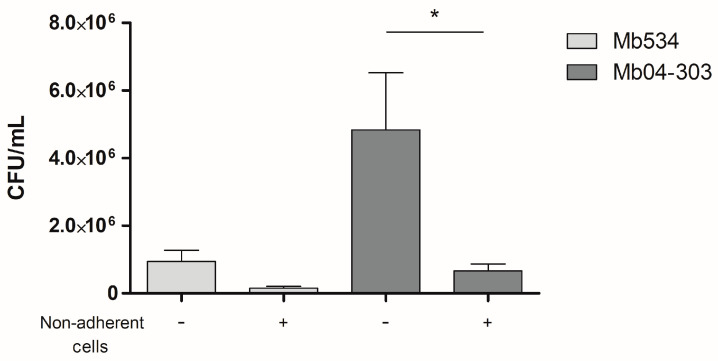
Mycobacterial growth inhibition assay. Macrophages were infected with *M. bovis* strains (MOI = 1) and co-cultured with autologous lymphocytes or culture media alone (control condition). Colony forming units were counted 3 h post infection (to detect any variation in inoculum between strains) and at 72 h (depicted in the graph). Cells were purified from three different calves (*N* = 3). Infections were carried out in parallel and in triplicate for each condition (technical replicates); the bars and whiskers represent mean values and SEM, respectively. * *p* < 0.05 ANOVA test with Bonferroni post-test for multiple comparisons.

Using an RNA-seq analysis, we compared the gene expression profiles of bovine PBMC containing macrophages infected with Mb04-303 and Mb534. For that purpose, macrophages differentiated from PBMC of healthy calves were infected with the studied strains, and co-cultured with the autologous lymphocyte fraction (see Section 2.4). In parallel, co-cultures from the same animals were used to quantify CFU, and confirm the inhibition of replication observed previously in Mb04-303 infected co-cultures (Figure 1). For the RNA-seq analysis, we selected samples from three different animals infected with Mb04-303 or Mb534 that met this criterion. We have previously demonstrated that in the absence of non-adherent cells, such as lymphocytes, infected macrophages failed to control the intracellular replication of Mb04-303 [4]. For this reason, the transcriptional analysis of infected macrophages cultured alone was excluded from this study.

**Figure 2:**
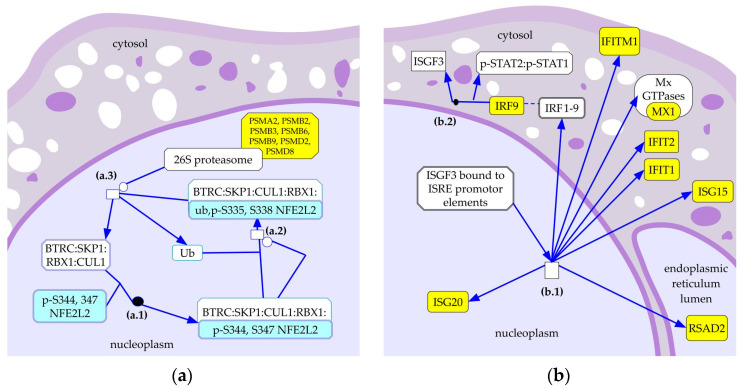
Differentially expressed biological pathways between infections with Mb04-303 and Mb534. (**a**) Downregulated and (**b**) upregulated pathways in Mb04-303 infections compared to Mb534 infections, respectively. Unregulated and downregulated genes in Mb04-303 are in yellow and light blue, respectively. Genes that did not modify their expression between conditions are in white. Differentially expressed pathways were identified using the Reactome database (https://reactome.org/ (accessed on 2 March 2023)). (**a.1**) BTRC binds p-S344, 347 NFE2L2, (**a.2**) p-S344, 347 NFE2L2 is ubiquitinated by BTRC:SKP1:CUL1:RBX1, (**a.3**) Ub,pS335,S338 NFE2L2 is degraded, (**b.1**) expression of IFN-induced genes, (**b.2**) interaction of IRF9 with p-STAT2:p-STAT1.

The analysis of RNA-seq data yielded 238 differentially expressed (DE) genes with a cut-off adjusted *p*-value of <0.25 (Table S2). Co-cultures infected with Mb04-303 presented 97 of the total downregulated DE genes and 141 upregulated genes when compared with the Mb534-infected co-cultures (Table S2). According to an analysis using the Reactome database, a curated database of pathways and reactions (https://reactome.org/ (accessed on 2 March 2023)), the Interferon alpha/beta signalling, including *ISG20*, *PTPN1*, *IFITM1*, *RSAD2*, *MX1*, *ISG15*, *IRF9* and *IFIT2* (Figure 2), was the most upregulated genes pathway in Mb04-303 infections. Reactome also identified the KEAP1-NFE2L2 pathway as differentially expressed between infections (Figure 2). Within this pathway, NFE2L2, which encodes a proteasome-regulated transcriptional factor involved in antioxidant genes, was downregulated, while the genes encoding the proteasome components PSMB2, PSMB3, PSMA2, PSMD2, PSMD8, PSMB6 and PSMB9 were upregulated in Mb04-303 infections.

These results indicate that Mb04-303-infections display an upregulation of the type 1 IFN signalling and a downregulation of the KEAP1-NFE2L2 pathway, compared to the Mb534-infections.

Other genes downregulated in the infections with Mb04-303 were *KMO*, *SKA1*, *ZNF367*, *SPRED1* and *HNRNPLL* (Table S2). KMO is an interferon type 1-induced gene [16], whereas HNRNPLL is a master regulator of activation-induced alternative splicing in T cells [17], and SPRED1 downregulates IL-33-mediated ERK activation and apoptosis in bone marrow group 2 innate lymphoid cells of mice [18]. Although *KMO*, *SPRED1* and *HNRNPLL* encode components of immune mechanisms, in this study, these genes did not integrate DE gene-network pathways. Thus, it is difficult to evaluate the impact of their downregulation on Mb04-303 infections.

ZNF367 participates in the regulation of apoptosis in spermatogonial stem cells [19], whereas SKA1 is part of an essential mitotic component required for an accurate cell division in human cells [20]. These two proteins are not directly involved in immune mechanisms; therefore, their role in the innate response induced in PBMC by Mb04-303 is even more uncertain.

### 3.2. Mb04-303 Induces More Damage in Endosomal Cell Membranes Than Mb534

**Figure 3:**
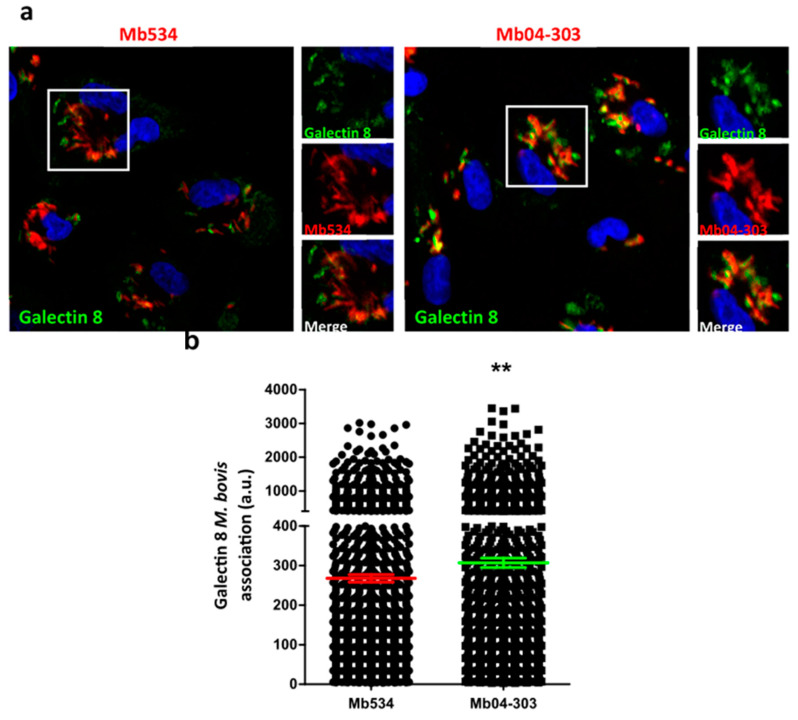
Mb04-303 induces more damage in endosomal cell membranes than Mb534. (**a**) Bovine macrophages were infected with Mb04-303-RFP (red) and Mb534-RFP (red) for 3 h of uptake, followed by 24 h of chase. The cells were fixed and subjected to indirect immunofluorescence using an antibody against Galectin 8 (green). Nuclei were visualised with TOPRO-3 (blue). The white squares outline the zoomed area shown in the insets. Scale bars: 10 µm. (**b**) Quantitative analysis of Galectin 8 association to the *M. bovis* strains after 24 h of infection in arbitrary units (a.u.). Data represent the Mean ± S.E.M of three independent experiments, (**) *p* ≤ 0.05, analysed with two-tailed Student’s *t*-test.

Phthiocerol dimycocerosates (PDIMs), together with ESX-1-secreted proteins, enable the permeabilization of the phagosome containing *M. tuberculosis* and thus facilitate the induction of the type 1 IFN response in infected macrophages [21]. Given that Mb04-303 produces more PDIM than the Mb534 strain [22], we next assess whether the triggering of IFN type 1 pathway activation in Mb04-303 infections was due to the release of bacterial compounds into the cytoplasm of macrophages. The evaluation of the recruitment of Galectin 8, which is a lectin that senses phagosomal damage [23], to phagosomes containing *M. bovis* strains revealed that this lectin colocalised slightly more with phagosomes containing Mb04-303 than with those containing Mb534 (Figure 3).

This result indicates that Mb04-303 causes more damage to the membrane of the endosomes than Mb534.

Altogether, the greater ability of Mb04-303 to perturb cell membranes, possibly due to the higher PDIM content, may favour the release of compounds, such as bacterial DNA, into the cell cytoplasm and this would trigger the activation of type 1 IFN signalling pathways.

### 3.3. ESAT-6 and CFP-10 Were Not Involved in the Transcriptional Signature Upregulated in Mb04-303 Infections

The ESX-1 type VII secretion system secretes the virulence factors ESAT-6 and CFP-10, among other proteins [23,24]. As ESAT-6 has been reported to drive the type 1 IFN response in *M. tuberculosis* and *Mycobacterium marinum* macrophage infections [24,25], we examined the impact of ESAT-6 and CFP-10 on the upregulation of type 1 IFN signalling pathways in co-cultures infected with Mb04-303. An RT-qPCR analysis of the transcripts of the target genes (Table S2) in macrophage co-cultures infected either with Mb04-303 mutant in the *esxA-esxB* genes (Mb04-303ΔesxA-esxB) or Mb04-303 wild type demonstrated that none of the selected genes showed differential expression between infections (Figure S2). This result indicates that ESAT-6 and CFP-10 did not drive the activation of the type 1 IFN signalling pathway observed in this study.

## 4. Discussion

In this study, we compared the transcriptional profiles of bovine macrophages infected with two *M. bovis* strains of varying virulence, and co-cultured with autologous mononuclear cells. An analysis of DE genes revealed that infections with Mb04-303, the virulent strain, led to the upregulation of the expression of IFN-inducible genes and the downregulation of the expression of genes associated with stress responses, when compared to the attenuated Mb534 strain.

Among the downregulated genes in Mb04-303 infections, the *NFE2L2* gene encodes a major transcription factor that responds to oxidative stress and inflammation through the induction of various antioxidant response elements. Under oxidative stress, NFE2L2 translocates to the nucleus, and upregulates the expression of genes that contribute to NLRP3 inflammasome activation [26]. However, depending on the context, NFE2L2 may have opposite effects on NLRP3 inflammasome activation [26,27].

In turn, NFE2L2 is regulated by a mechanism that involves its degradation via proteasome through the ubiquitin-dependent pathway [28,29]. In line with the *NFE2L2* downregulation, Mb04-303 infections upregulated the expression of *PSMB2*, *PSMB3*, *PSMA2*, *PSMD2*, *PSMD8*, *PSMB6* and *PSMB9*. All these genes encode for components of the proteasome complex.

The activation of the inflammasome complex is relevant for host defence against pathogenic mycobacterium species [30]. In particular, interleukin-1β (IL-1β), a critical regulator of the inflammatory response, is secreted by a mechanism that depends on the inflammasome activation. Notably, IL-1β and prostaglandin E2 are antagonists of type 1 IFN in *M. tuberculosis* infection [31]. Consistent with the role of IL-1β in the inhibition of IFN production, in this study, Mb04-303 infections upregulated the expression of genes stimulated by IFN (ISG). The database, Reactome, identified IFN α/β signalling genes upregulated in infections with Mb04-303, when compared to Mb534 infections.

Virulent strains of *M. tuberculosis* induce high levels of type 1 IFN in vivo both in humans and mice [32], and the high levels of this cytokine have a negative effect on the protection against *M. tuberculosis* and *M. bovis* [33,34,35]. Aguilar de Leon et al. have observed that, unlike infections with Mb534, Mb04-303 caused sudden pneumonia with extensive necrosis lesions and high bacterial loads in the lungs of BALB/c mice intratracheally inoculated [36]. Thus, the comparative transcriptomic profile of Mb04-303 and Mb534, which shows higher expression of IFN α/β signalling genes in Mb04-303 infections, is consistent with the differential virulence of both *M. bovis* strains. In line with the results of this study, Jensen et al. have found that the *M. bovis* strain that better survived and replicated in bovine macrophages induced higher levels of type 1 interferons [37].

In opposition to the deleterious effect of type 1 IFNs in controlling tuberculosis infections, these cytokines activate myeloid cells to enhance the immune response during bacterial infection (reviewed in [31]). In particular, the study of Swaim et al. has shown that ISG15 enhances IFN-γ secretion by NK cells [38]. Interestingly, in our research, an ISG was upregulated in Mb04-303 infections. The action of ISG15 observed by Swaim et al. on NK cells may explain, in part, the effect of PBMC on the control of the Mb04-303 replication inside macrophages (Figure 1).

Therefore, the results of this study support the concept that differences in temporal and spatial induction of type 1 interferons during *M. bovis* and *M. tuberculosis* infections determine their beneficial or detrimental effects on the control of these pathogens (reviewed in [31]). The observed opposite effects of the type 1 IFN action also occur in other intracellular pathogens (reviewed in [39]).

The context-dependent dual role of type 1 IFN reconciles the fact that macrophages infected with Mb04-303 cross-talk with other immune cells to induce a strong innate response capable of controlling intracellular replication of mycobacteria [4]. However, eventually, this highly virulent strain would manage to overcome the microbicidal mechanisms of the host by manipulating the immune response in its favour to finally establish a severe disease [36]. Likely, an overstimulation of type 1 IFN signalling upon the infection of co-cultures with Mb04-303 eventually results in the suppression of the immune responses, a phenomenon widely documented in viral infections [40]. Further transcriptional analyses of long-term infections with Mb04-303 are necessary to confirm this presumption.

Two reports have suggested that PDIM and the ESX-1 secretion system act in concert to produce phagosomal permeabilisation [21,41]. In addition, this membrane permeabilisation allows mycobacterial DNA to access host cytosolic receptors and, therefore, the interaction with these receptors activates the type 1 IFN response [21,42]. Consistent with the requirement of a membrane damage for a successful activation of type 1 IFN response, Mb04-303 infections produced higher membrane damage in phagosomes than Mb534 infections in this study, as determined with Galectin 8 recruitment on phagosomes containing mycobacteria.

In addition, the mechanisms deployed by *M. tuberculosis* and *M. marinum* to persist and replicate in macrophages require ESX-1-driven type 1 IFN-signalling [24,25]. In this study, however, the upregulation of ISGs upon infection with the Mb04-303 strain was independent of ESAT-6 and CFP-10. Depending on the context, the binding of type 1 IFN to the IFNα receptor (IFNAR) produces differential activation of signal transducer and activator of transcription (STAT); this differential activation would control distinct gene-expression programmes which, in turn, can exert disparate biological functions (reviewed in [43]). In fact, in this study, Mb04-303 infections induced the IRF9 pathway, while ESX-1-mediated type 1 IFN-signalling seems to have occurred via the IRF3-dependent pathway [24].

Altogether, these results suggest that the cellular events that led to the activation of the type 1 interferon pathway in Mb04-303 infections involved phagosomal membrane disturbance, although this mechanism seems to be independent of the action of ESAT-6 and CFP-10, as previously proposed. Possibly, other ESX-1 secretion system proteins still present in the Mb04-303ΔesxA-esxB strain disrupted the phagosomal membrane with the assistance of PDIMs.

In conclusion, while Mb04-303 infection upregulated the interferon-mediated signalling pathway, when compared to infections with the more attenuated Mb534 strain, it downregulated the pathway mediated by the inflammasome. In addition, the results of this study support previous findings where type 1 IFN suppressed IL-1 production through STAT1-dependent inhibition of NLRP3 and NLRP1 inflammasome activity [44].

While the results of this study are limited to the early events of the interaction between *M. bovis* strains and the naïve bovine immune cells, the findings suggest that a genetically manipulated Mb04-303 strain (so as to eliminate its virulent capacity) could be a promising vaccine candidate for controlling bTB.

## Data Availability

Data sets of RNAseq were deposited in GEO Submission (GSE241059).

## Supplementary Materials

The following supporting information can be downloaded at https://www.mdpi.com/article/10.3390/pathogens12091159/s1, Figure S1: Absence of ESAT-6 in Mb04-303ΔesxA-esxB. Figure S2: Gene expression fold-change differences between co-cultures infected with Mb04-303 (*n* = 4–6) and Mb04-303ΔesxA-esxB (*n* = 4–6) using RT-qPCR. Table S1: Primers used in this study. Table S2: Differentially expressed genes between infections with Mb04-303 and Mb534.
