# Supplementary material for: A Transcriptional Analysis of Cattle Immune Cells Reveals a Central Role of Type 1 Interferon in the In Vitro Innate Immune Response against Mycobacterium bovis
Source: Pathogens. 2023 Sep 14;12(9):1159. doi: 10.3390/pathogens12091159 (PMC10536033; doi:10.3390/pathogens12091159)
Supplement: Supplementary file 1 [file pathogens-12-01159-s001.zip › Supplementary material.pdf]

Supplementary material

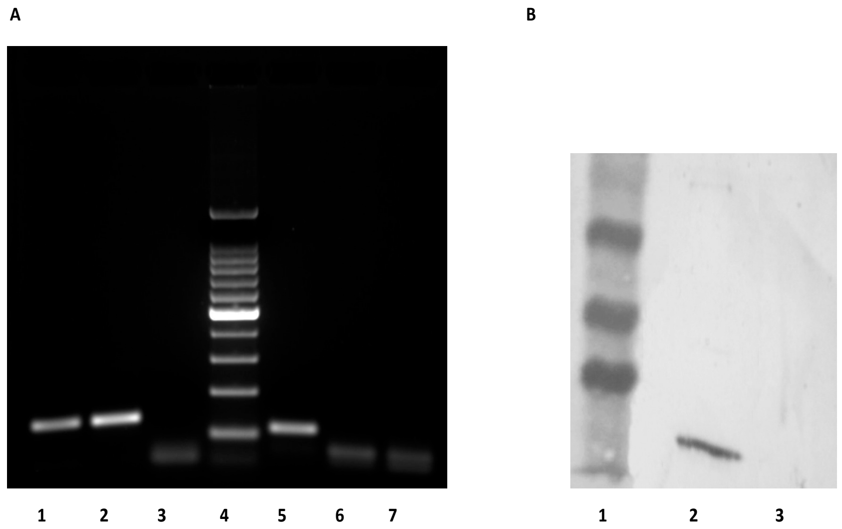

Figure S1

Absence of ESAT-6 in Mb04-303ΔesxA-esxB. (**A**) Agarose gel electrophoresis of PCR amplification products using primers that hybridise in *sigA* (lines 1 to 3, 135 bp) and *esx-A* (lines 5 to 7, 110 bp) gene sequences. Mb04-303 or Mb04-303ΔesxA-esxB DNA were used as template for the PCR reactions in lines 1 and 5 and lines 2 and 6, respectively. Negative controls were included in lines 3 and 7. Line 4: 100 bp marker (Inbio Highway, Argentina). (**B**) Western blot of 2: Mb04-303, 3: Mb04-303ΔesxA-esxB developed with monoclonal anti-ESAT-6 as primary antibody. Line 1: pre-stained marker (Blue Plus Protein Marker, Transgenbiotech, Beijing, China). The dotted arrows indicate the position of the monomeric ESAT-6 protein.

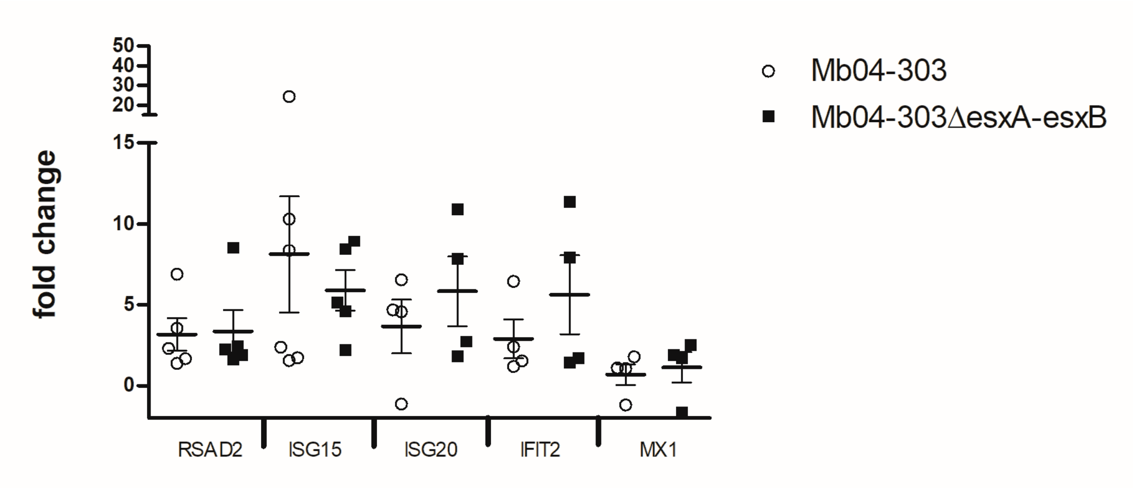

Figure S2

Gene expression fold-change differences between co-cultures infected with Mb04-303 (n =4-6) and Mb04-303ΔesxA-esxB (n = 4-6) using RT-qPCR.

Relative gene expression was calculated using the 2-ΔΔCt method with E correction, using *gadph* mRNA expression as reference gene and the non-infected co-culture condition as the calibrator. Statistical differences between fold change of expression for each of the selected genes in Mb04-303 and Mb04-303ΔesxA-esxB infected co-cultures were analysed using Mann-Whitney test and Grubbs test to exclude outliers (non-significant differences were observed for the five genes evaluated).
