# Supplementary material for: A Transcriptional Analysis of Cattle Immune Cells Reveals a Central Role of Type 1 Interferon in the In Vitro Innate Immune Response against Mycobacterium bovis
Source: Pathogens. 2023 Sep 14;12(9):1159. doi: 10.3390/pathogens12091159 (PMC10536033; doi:10.3390/pathogens12091159)
Supplement: Supplementary file 1 [file pathogens-12-01159-s001.zip › Table 1S.pdf]

Table S1

Primers used in RT-qPCR

| | |
|---|---|
| RSAD2-up | GACTGGACCTGGCTTTTCTG |
| RSAD2-low | GGCTCTGGAACTTTCTCACG |
| ISG15-up | AGCGTGTACAAGCAGACCAG |
| ISG15-low | ACCCCGAAGACGTAGATTCA |
| ISG20-up | AGCTACTGAGCTCCCGACAG |
| ISG20-low | GGGAATCTGACCCTGTTTCA |
| IFIT2-up | CAGGAGGCAGAGAGACATCC |
| IFIT2-low | AGAAACTGGCAAGGCAAAGA |
| MX1-up | CCCAAGGAGACAGGACCATA |
| MX1-low | TAGCCCTTCTTCAGGTGGAA |

Primers used for construction of Mb04-303ΔesxA-esxB mutant strain

| | |
|---|---|
| (I) MutesxA/B-low | GCGGCCGCTCGATCTGGGTTTTCAGGTC |
| (II) MutesxA/Bphage-up | CGATCGCGGCTCCACCAGGCCGGG |
| (III) MutesxA/Bphage-low | CGATCGCGGCTGCGCTGCCATATC |
| (IV) MutesxA/B-up | AAGCTTATGTTCGCATAGGGCAACG |

Primers used for confirmation of *esxA-esxB* deletion in Mb04-303ΔesxA-esxB

| | |
|---|---|
| SigA-up | GGCCAGCCGCGCACC |
| SigA-low | GTCCAGGTAGTCGCG |
| esat6-up | ACGTCCATTCATTCCCTCCTT |
| esat6-low | TCCCATTTTTGCTGGACACC |
